# Hyperparathyroidism and the Hematologist

**DOI:** 10.1002/ajh.27527

**Published:** 2024-11-12

**Authors:** Hajer Oun, Kirsteen Harper, Mike Leach, Barbara J. Bain

**Affiliations:** ^1^ Department of Haematology Beatson West of Scotland Cancer Centre Glasgow UK; ^2^ Centre for Haematology, Department of Immunology and Inflammation St. Mary's Hospital Campus of Imperial College London School of Medicine London UK

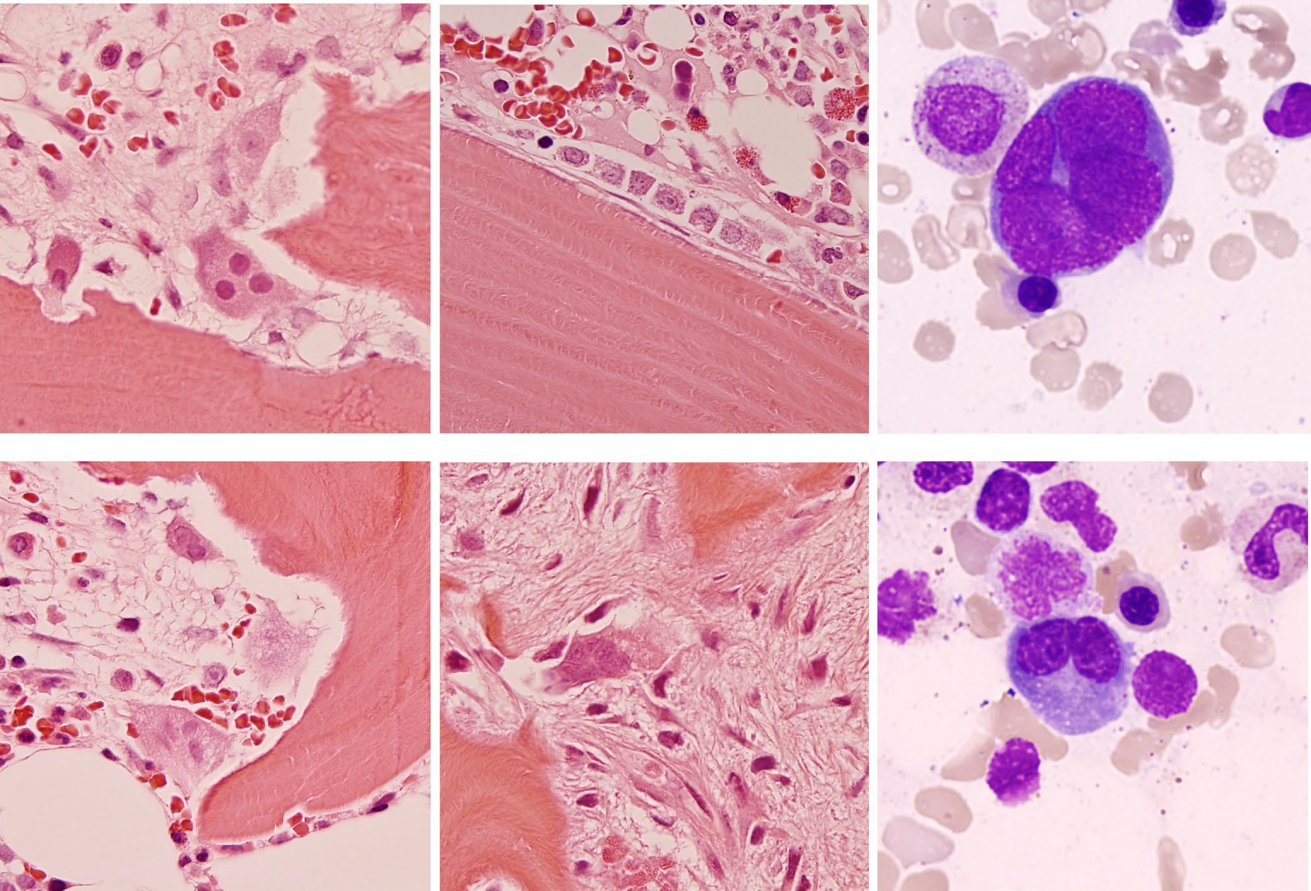

A 76‐year‐old man with a history of chronic obstructive pulmonary disease with lung fibrosis, type 2 diabetes mellitus, and chronic kidney disease underwent computed tomography imaging of the chest due to increasing dyspnea. The bones appeared sclerotic, and a bone scan showed diffuse tracer uptake throughout the axial and appendicular skeleton. The prostate showed no features of malignancy on magnetic resonance imaging and prostate‐specific antigen was 6.6 μg/L (normal range (NR) 0–5). Serum tryptase levels were mildly elevated at 16 μg/L (NR 2–14) on two occasions. Biochemical investigations showed vitamin D < 14 nmol/L (NR > 50), alkaline phosphatase 665 U/L (NR 30–130), parathyroid hormone 52.8 pmol/L (NR 1.6–7.5), calcium 2.43 mmol/L (NR 2.2–2.6) and phosphate 1.07 mmol/L (NR 0.8–1.5), in keeping with hyperparathyroidism secondary to vitamin D deficiency and chronic kidney disease (creatinine 169 μmol/L and estimated glomerular filtration rate 34 mL/min).

Bone marrow trephine biopsy sections showed areas of active bone resorption by multinucleate osteoclasts forming recesses known as Howship's lacunae (top and bottom left, all histological images hematoxylin and eosin, ×50 objective). In other areas, lamellar bone was being actively laid down by rows of osteoblasts (top center). There was patchy fibrosis at sites of previous bone resorption (bottom center). Notably, there were osteoclasts also visible in the marrow aspirate (top and bottom right, May–Grünwald–Giemsa, ×100 objective). There was no abnormal mast cell population.

These features are typical of hyperparathyroidism where osteoclasts strive to release calcium whilst osteoblasts attempt to repair the trabecular damage. This active bone remodeling with the associated trabecular changes generates the sclerotic radiological appearance of the affected bones. Osteoblasts and osteoclasts normally work together in bone repair, remodeling, and growth but this process is exaggerated under the influence of increased parathyroid hormone whether primary, due to a parathyroid adenoma, or secondary, as a result of vitamin D deficiency or chronic kidney disease. The recognition of the features of bone disorders with associated bone marrow fibrosis is important so that they are not confused with myeloproliferative neoplasms.

## Conflicts of Interest

The authors declare no conflicts of interest.

